# Prediction of Breast Cancer from Imbalance Respect Using Cluster-Based Undersampling Method

**DOI:** 10.1155/2019/7294582

**Published:** 2019-10-16

**Authors:** Jue Zhang, Li Chen, Fazeel Abid

**Affiliations:** ^1^School of Information Science and Technology, Northwest University, Xi'an 710127, China; ^2^School of Information Engineering, Yulin University, Yulin 719000, China

## Abstract

To overcome the two-class imbalanced problem existing in the diagnosis of breast cancer, a hybrid of K-means and Boosted C5.0 (K-Boosted C5.0) is proposed which is based on undersampling. K-means is utilized to select the informative samples near the boundary. During the training phase, the K-means algorithm clusters the majority and minority instances and selects a similar number of instances from each cluster. Boosted C5.0 is then used as the classifier. As there is one different instance selection factor via clustering that encourages the diversity of the training subspace in K-Boosted C5.0, it would be a great advantage to get better performance. To test the performance of the new hybrid classifier, it is implemented on 12 small-scale and 2 large-scale datasets, which are the often used datasets in class imbalanced learning. The extensive experimental results show that our proposed hybrid method outperforms most of the competitive algorithms in terms of Matthews' correlation coefficient (MCC) and accuracy indices. It can be a good alternative to the well-known machine learning methods.

## 1. Introduction

Breast cancer is one of the top ten causes of women death around the world [1]. Until now, the cause of breast cancer has been still under research, and the most effective treatment depends on the time when the cancer is detected. Now, early detection is the only way to ensure long survival of the patients [2, 3], which means, if the breast cancer is detected timely, the chance of patient survival is increased. Therefore, accurate diagnosis of breast cancer has become one of the challenging problems in the medical science community.

There has been a great deal of research on medical breast cancer diagnosis in the literature, and many of them gained high classification accuracies. Li et al. [4] presented a novel supervised feature extraction method called quasiconformal kernel common locality discriminant analysis (QKCLDA) to map the input data to a low space, and the obtained classification accuracy was 97.26%. A hybrid of K-means and SVM (K-SVM) algorithm was proposed by Zheng et al. [5], and the obtained accuracy was 97.38%. Pashaei et al. [6] used a combination of particle swarm optimization with boosted C5.0 decision tree classifier (PSO + Boosted C5.0) and reported the accuracy of 96.38%. Weng et al. [7] applied a multiple neural network classifiers (EC) technique to the breast cancer diagnosis and achieved an accuracy of 96.5%. Pashaei et al. [8] proposed a novel dimension reduction method named as binary version of Black Hole Algorithm (BBHA), which obtained 97.38% accuracy. A knowledge-based system using the fuzzy logic method (referred to as EM-PCA-CART-Fuzzy Rule-Based) was developed by Nilashi et al. [9] to increase the predictive accuracy of breast cancer disease classification. They improved the accuracy to 93.2% accuracy. All these methods were evaluated on Wisconsin Diagnostic Breast Cancer Dataset (Wbcd). Peng et al. [10] incorporated artificial immune into semi-supervised learning for unlabeled breast cancer diagnosis data (referred as Aisl). They obtained 98% and 98.3% accuracies on Wbcd and Breast Cancer Wisconsin (Bcwo) datasets, respectively. In 2016, a new model for determination of kernel bandwidth based on the particle swarm optimization (PSO) method with nonparameter kernel density estimation (KDE) was proposed by Sheikhpour et al. [11], and the obtained accuracies were 98.45% and 98.53%, respectively. Wang et al. [12] proposed a SVM-based weighted AUC ensemble learning model for breast cancer diagnosis on Wbcd and Bcwo datasets and achieved accuracies of 97.68% and 97.10%, respectively. In summary, all these methods show an improvement in accuracy in breast cancer diagnosis, but a defect was noted that these methods aimed to maximize accuracy and used the training accuracy as the only criterion to evaluate the performance, and this is based on the assumption of balanced dataset. But in a real application, the balanced dataset assumption of medical diagnosis is frequently violated, since the examples of the noncancer class outnumber the examples of the cancer class.

Imbalance problem should be carefully addressed because traditional methods are designed to maximize the global accuracy, but exhibit poor generalization for the small class which is usually the most primary one. Thus, for the traditional algorithm, rare class is difficult to identify than the majority class. Hence, the breast cancer diagnosis problem should be classified from the perspective of class imbalance.

The popular mechanism to address the problem of class imbalance is the ensemble of classifiers with a data-based approach since the data-based method and classifier training task can be performed independently [13, 14]. For a data-based approach, oversampling and undersampling are the most useful solutions. In the first case, some literature agrees that this can increase the probability of overfitting [15]. Undersampling has been proven to be better than oversampling, but it suffers from high elimination of useful samples [16].

To overcome the limitation of undersampling, a K-means clustering-based undersampling method is employed to select the samples near the boundary since the border samples are the most informative ones and play an important role in the classification [17–19], thereby preserving the maximum of useful samples. Meanwhile, we can adjust the strength to reconstruct small size of subset for training by boosted C5.0, which has been considered as the most effective algorithms for breast cancer diagnosis [14, 15, 20]. The objective of this paper is to discuss the clustering-based undersampling method for training boosted C5.0 for class imbalanced data, especially breast cancer prediction data, and we will mainly focus on the undersampling strategy.

The major contributions of this paper are: (1) a strategy of using the k-means clustering technique for undersampling both majority and minority classes are presented, (2) an efficient classifier ensemble is considered. Boosting scheme is used to leverage the strength of base classifier, (3) an extensive experiment analysis is carried out on 12 real imbalanced data sets, showing that the proposed method K-Boosted C5.0 can outperform the results of the literature and state-of-the-art methods, RUSBoost and SMOTEBoost which are renowned methods in this area and hence proving the inherent advantage of the proposed approach.

The remainder of this paper is organized as follows.  Section 2 describes the research methodology including instance selection method and model construction. The evaluation metrics and experimental results are presented in Section 3.  Section 4 presents a discussion. Finally, Section 5 concludes the paper and the indication for the intended direction of future research.

## 2. Materials and Methods

**Figure 1:**
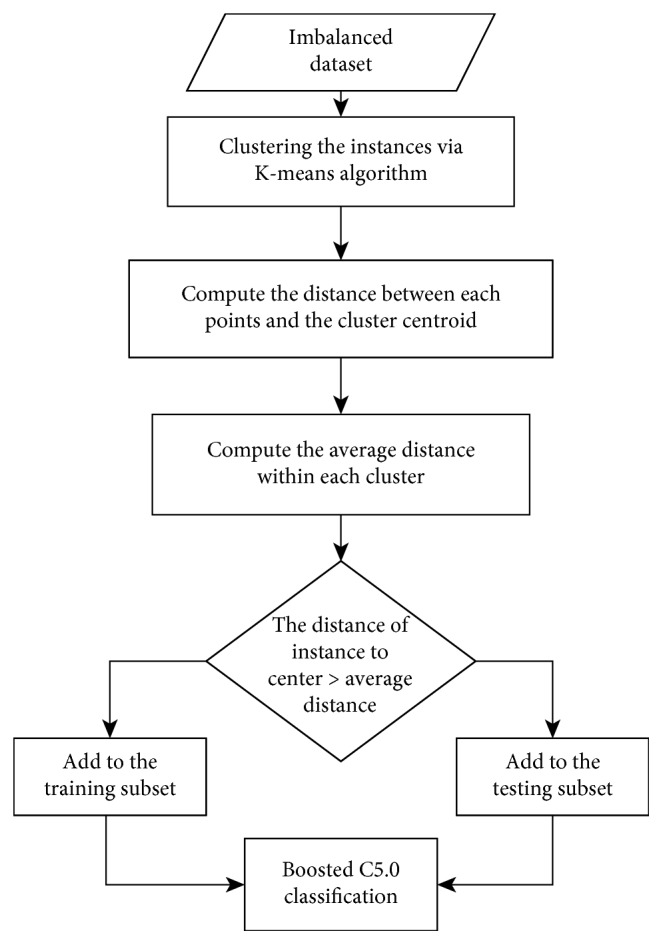
Block diagram for the proposed classification model.

In this section, we give a detailed description of the K-means clustering-based undersampling algorithm. The processes are shown in Figure 1. The clustering-based undersampling method is employed to select the border samples in the majority and minority classes. The obtained samples are combined together, and a balanced training subset is obtained. The balanced training subset is used as the input to the boosted C5.0 classifier.

### 2.1. Clustering-Based Undersampling Method

Undersampling is a better choice than oversampling since the oversampling method increases the likelihood of overfitting; however, undersampling also suffers from the problem of underfitting, in other words, useful data might be eliminated. To overcome the limitation, a clustering-based undersampling method is proposed. As described in the aforementioned literature, interior prototypes can be discarded since they have little effect on classification accuracy, but the border prototypes are critical with emphasized impact for classification, that could be important for the induction process. Thus, in our proposed method, we use the clustering-based undersampling method to select the samples near the boundary region to rebalance the class distribution without significant loss of classification accuracy. The aim of clustering is to group objects into two clusters. Thus, we can select the optimal samples which lie to the cluster boundary, resulting in a balanced dataset with each cluster containing similar number of data. The idea behind this implementation of clustering-based undersampling is to eliminate the examples from both classes that are distant from the cluster border since these kinds of examples might be considered less relevant for learning. In this paper, only the K-means clustering algorithm is considered because it is simple and efficient [21].

This proposed clustering-based undersampling method has three stages: firstly, clustering the overall samples via K-means algorithm. Secondly, compute the distance from each point to the cluster centroid. Finally, the sample whose distance to the central point is greater than the cluster average distance is merged, resulting in a modified balanced data set. The remaining samples are used as the testing subset. We calculate the distance using Euclidean distance. The number of clusters was set to two for binary-class datasets. It should be pointed out that if the obtained subset is an imbalance dataset after performing instance selection algorithm, we should change the selection condition that the samples whose distance to the cluster central points are greater than half of the average distance of the clusters are selected and added to the training space. The details of the informative sample selection algorithm are described below. The pseudocode presented in Algorithm 1 describes the clustering-based undersampling algorithm in detail.

Step 1: randomly select *k* sample instance from *X* as the centroids point in the cluster, *k* is determined by the number of clusters.
Step 2: Euclidean metric is used for computing the distance between each point and the centroid in the same cluster, and each data point is assigned to its closest centroid. The distance between *x*_*i*_ and the point *u*_*k*_ is defined by the following equation:
  (1) $$\begin{array}{c} J\left(c_{k}\right)=\underset{x_{i}\in c_{k}}{\sum }\left\|x_{i}-u_{k}\right\|. \end{array}$$
Step 3: compute a new cluster centroid point for reducing the Euclidean distance.
Step 4: repeat steps 3 and 4 until cluster membership stabilizes.
Step 5: compute the average distance of each number *i* to the centroid *u*_*k*_ in the same cluster *s*_*k*_, which is calculated by using the following equation:
  (2) $$\begin{array}{c} d_{\text{avg}}=\frac{\sum_{k=1}^{K}\sum_{i\in S_{k}}\sqrt{\sum_{j=1}^{D}{\left(X_{j}^{i}-X_{j}^{\mu_{k}}\right)}^{2}}}{N}. \end{array}$$
Step 6: create the final training data set 〈*X*′〉, by appending the point *x*_*i*_ to *X*′ if *J*(*c*_*k*_)≺*ηd*_avg_.

When the clustering-based undersampling method is employed, the redundant samples can be removed, the scale of samples has been greatly reduced, and the basic information of the original database can be retained. In this case, the time and space complexity of the algorithm can be reduced, and the classification accuracy can be improved.

### 2.2. Boosted C5.0

C5.0 is an improved algorithm from C4.5 by Ross Quinlan, and the test attributes by information gain [22], which has a noticeably lower error rate and uses an order of magnitude less memory. It creates a decision tree model in the way of “divide and rule” and prunes the tree by postpruning algorithm. All the nodes are divided into their class until the nodes cannot be divided. In addition, the most worthless in the low splits of the tree is removed or pruned.

Boosting is the most commonly used technique in the imbalance framework for constructing ensembles. The boosting algorithm repeatedly calls weak learner, each time feeding it a different distribution over the training data. C5.0 can easily support for boosting, and the boosting technique can improve the performance. The class imbalance can be considered seriously in medical decision making and boosted C5.0, which has been widely considered, being now a well-know method on imbalance learning. Thus, in this paper, we come up with boosted C5.0 to make use of the advantage and avoid the shortcoming.

## 3. Results and Discussion

To clearly observe the impact of clustering-based undersampling on imbalance dataset and investigate how the performance measures behave along with the clustering-based undersampling degree in depth, we develop ensembles on imbalanced data sets with different degrees of class imbalance. In our experiments, the items to be investigated are as follows: (a) the ability of keeping majority classification accuracy and (b) the ability of improving the minority classification accuracy. The experiments are performed by using a laptop with Windows 10, 2.19 GHz Pentium CPU and 4 GB RAM, using Matlab version 2016a and R version 3.4.4. Additionally, boosted C5.0, naïve Bayes and SVM classifiers, “C50” and “e1071”, and “kernlab” packages have been used accordingly. For 10-CV algorithm, the “caret” package has been utilized. All packages with default setting were used.

In our experiments, *k* is set to 2 since all classification tasks are two-class imbalance cases. As for boosted C5.0, we use 10 trails for boosting. For the SVM classifier, the linear kernel function is used to avoid overfitting as the most formative samples have been selected as the training set.

### 3.1. Dataset

The work in this paper confers four experimental studies. In the first study, 2 small-scale breast cancer datasets are used. In the second study, 10 small-scale datasets from UCI repository with various imbalance rates and data set sizes were used. They are all two-class classification datasets, and if the instances contained in the datasets have the missing values in different attributes, they will be discarded from the datasets. These datasets are all representative ones that have been used in breast cancer diagnosis and imbalance learning in the literature. The third one was based on two large-scale datasets used by Lin et al. [23]. The fourth one discusses the computational time in seconds for each of the methods against each dataset. The data set information is summarized in Table 1.

### 3.2. Performance Measures

Overall accuracy becomes meaningless when the learning concern is how to find minority examples effectively [24]. Other performance criteria must be considered, and as pointed out by Raeder et al. [25], the choice of evaluation metrics plays an important role in imbalanced learning. However, some performance criteria such as *G*-mean, the area under curve (AUC), and F-measure are the commonly used ones in the class imbalance learning community. But, as suggested in the literature [26], if the classes are unbalanced, computing MCC is more appropriate than others since it represents the quality of unbalanced binary classification. The MCC is calculated according to the confusion matrix in Table 2 as follows:

(3) $$\begin{array}{c} \text{MCC}=\frac{\text{TN}\times \text{TP}-\text{FN}\times \text{FP}}{\sqrt{\left(\text{TP}+\text{FP}\right)\left(\text{TP}+\text{FN}\right)\left(\text{TN}+\text{FP}\right)\left(\text{TN}+\text{FN}\right)}}. \end{array}$$

In this study, the class of interest is known as the positive class, while all others are known as negative. Hence, the noncancer class is given “negative” and the cancer is given “positive.”

### 3.3. Experiment I

The data sources are taken from the breast cancer machine learning repository, which are Wbcd and Bcwo datasets. These are the complete and representative datasets. Thus, the testing results are reliable and valuable.

We compare the results with RUSBoost [27] and SMOTEBoost [28] which are all representative approaches combining resampling techniques with classifier ensemble. In addition, K-Boosted C5.0 is compared with SMOTE-Boosted C5.0 utilizing 10 fold-cross validation methods. The parameters of over and under in SMOTE algorithm are 100 and 300. The value of *η* is set to 0.25 and 1 in Wbcd and Bcwo datasets. Note that in the SMOTE-Boosted C5.0, we first generate data by SMOTE and then classify the samples by boosted C5.0 algorithms based on the 10 fold-cross validation sample selection method. These results are reported in terms of six measures: accuracy, sensitivity, specificity, *G*-mean, AUC, and MCC.

Table 3 reports the value of performance on each dataset, and the best performance is highlighted in bold typeface. In order to perform a comprehensive comparison of our proposed method, the comparison results using K-Boosted C5.0 in Wbcd and Bcwo datasets compare with different predicting methods in the literature listed in Tables 4 and 5.

For comparison purpose, the performance of K-Boosted C5.0 on Wbcd and Bcwo datasets is compared with that of other methods from the literature. Tables 4 and 5 report the results of K-Boosted C5.0 and different classification methods for Wbcd and Bcwo, respectively. The symbol “—” in Tables 4 and 5 indicates that we do not get the data from the reference.

From the results of Tables 4 and 5, one observed that K-Boosted C5.0 is significantly better than the results of the literature. For the Wbcd dataset, K-Boosted C5.0 has obtained 98.2% accuracy with 30 features. It should be pointed out that for the Wbcd dataset, a new method based on the modified correlation rough set FS and MLP classifier was used, and the obtained accuracy was 100%. In this method, only 3 features are obtained using an 80-20 train-test scheme [29]. These results show that the proposed model can achieve high classification performance when working with fewer feature variables, but in reality, it does so at cost of efficiency; in addition, the accuracies of K-Boosted C5.0 and FSMLP were not significantly different from each other on this dataset.

For the Bcwo dataset, it is evident from Table 5 that the proposed K-Boosted C5.0 has a higher MCC of 93.6%. When our algorithm is compared with the results of the literature, the results are similar in term of accuracy, but our method has almost perfect sensitivity, specificity, and *G*-mean. Therefore, it is possible to say that the proposed K-Boosted C5.0 algorithm performs as better as the state-out-method results of the literature. Note that K-Boosted C5.0 gets the higher accuracy and *G*-mean in two breast cancer datasets. These results illustrate the availability of choosing K-Boosted C5.0 as the classifier for breast cancer diagnosis. In order to further investigate the effectiveness of K-Boosted C5.0, we also provide some insight into clustering undersampling method and the MCC measure at different levels of imbalance rate.

### 3.4. Experiment II

In order to illustrate the generalization performance of the K-Boosted C5.0 method, our experiments are tested on ten data sets which are shown in Table 1. In these experiments, *η* is set to 0.5 in Yeast1 dataset, 0.7 in Redwine1 dataset, 0.8 in Redwine4 dataset, 0.9 in Abalone dataset, 1 in Yeast2 and Yeast3 datasets, 1.2 in Redwine2 dataset, 1.35 in Whitewine dataset, 1.5 in Pima dataset, and 1.6 in Redwine3 dataset. Especially, we have, respectively, selected ten different values (including 10, 3, 8, 4, 3, 1, 7, 1, 3, and 8) as the trial values in our ensemble method.  Table 6 provides the results of the experiments on ten imbalanced datasets, and the best MCC is highlighted in bold typeface.

**Figure 2:**
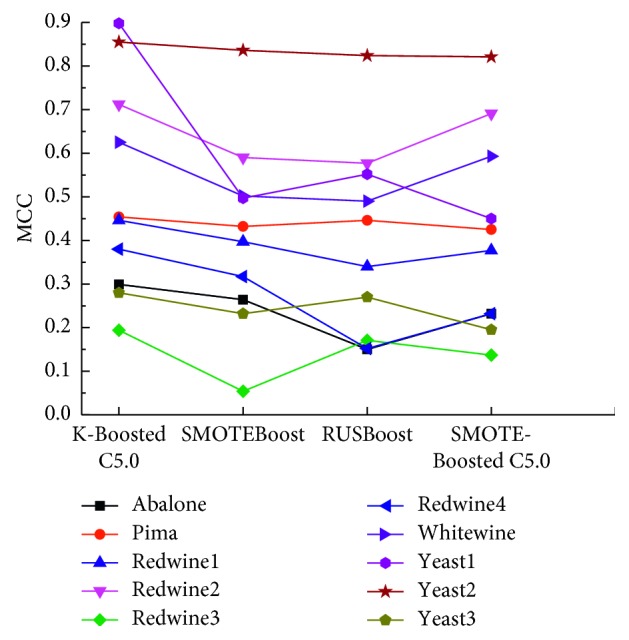
MCC result comparison based on different datasets.

The accuracy, sensitivity, specificity, *G*-mean, AUC, and MCC of four approaches on ten datasets are presented in Table 6. All the datasets are unbalanced. Thus, MCC is used to evaluate performance. In order to show the behavior of the K-Boosted C5.0 method, Figure 2 reports the value of MCC from the entire dataset by K-Boosted C5.0, SMOTEBoost, RUSBoost, and SMOTE-Boosted C5.0. As can be seen from Table 6 and Figure 2, it is clear that MCC of K-Boosted C5.0 is significantly better than all the other state-of-the-art methods, benefiting from the clustering-based undersampling technique.

### 3.5. Experiment III

**Figure 3:**
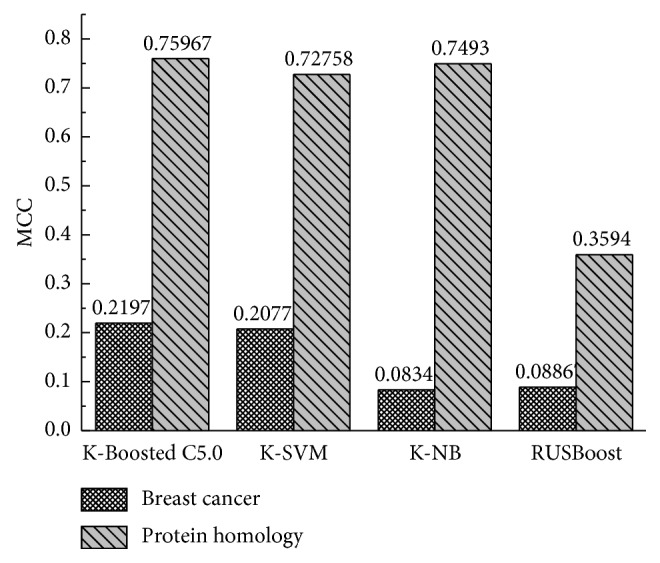
Classification of MCC of the different classifiers over the breast cancer and protein homology datasets.

In the third experimental study, for the clear observation of the impact of clustering-based undersampling on imbalanced data sets, two different classifiers were constructed, namely, the support vector machine (SVM) and naive Bayes (NB). In addition, in order to evaluate the performance of the proposed ensemble approach, RUS is used as the baseline for performance comparisons. As indicated by the performance results in Figure 3, the proposed clustering-based undersampling method combined with the boosted C5.0 ensemble classifier demonstrated the highest classification performance in terms of MCC over these two large-scale datasets. As it can be observed from the results listed, K-Boosted C5.0 has an outstanding performance, and the K-Boosted C5.0, K-SVM, and K-NB are significantly better than RUSBoost in these two large-scale datasets. This improvement in classification of MCC is mainly due to the clustering method. These results can lead us to conclude that the K-means clustering-based undersampling method can be effective to solve the imbalance problem for large-scale datasets that contain relatively large number of instances and imbalance rations. In addition, using the Boosting C5.0 ensemble method is preferable to using other traditional methods.

### 3.6. Experiment IV

**Figure 4:**
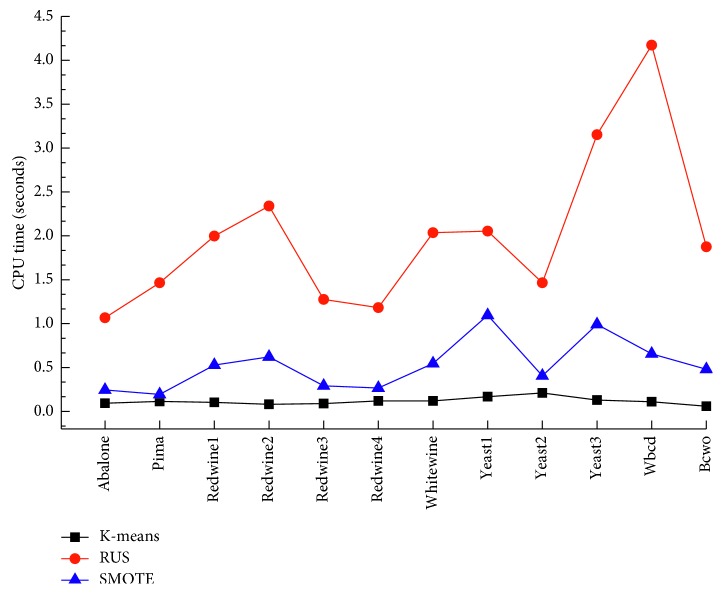
Computational efficiency of approaches on 12 datasets.

**Figure 5:**
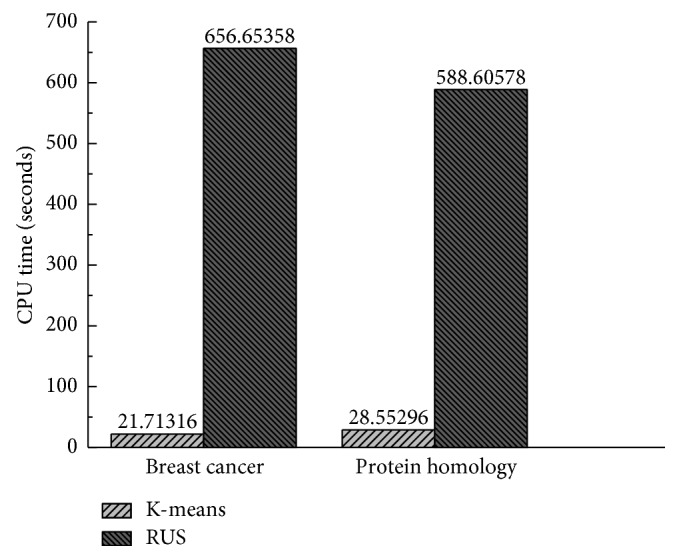
Computational efficiency of approaches on breast cancer and protein homology datasets.

In the fourth experimental study, the CPU time of the proposed method K-Boosted C5.0 was compared with the baseline algorithms, RUS and SMOTE, over ten small-scale datasets. In order to make the observation more convincing, the CPU time of the proposed method K-Boosted C5.0 was compared with RUS over two large-scale datasets.  Figure 4 shows the result obtained by K-means, RUS, and SMOTE sampling methods using the ten datasets.  Figure 5 shows the results obtained by K-means and RUS sampling methods using breast cancer and protein homology datasets, respectively. It is worth to note, for large-scale datasets, using SMOTE the CPU time is larger than one hour, since the long runtime is required by finding the nearest neighbor. The proposed K-means undersampling approach significantly outperformed all methods over ten small-scale and two large-scale datasets.

In order to confirm whether or not the comparative methods are significant, the Friedman test with 95% confidence level [30] is carried out. All the methods in ten small-scale datasets are sorted according to the mean ranks on their MCC performance measures since MCC is the accepted measure in class imbalance learning [26]. The alternative hypothesis is that there is no significant difference among these methods. Subsequently, Table 7 displays the *p*-value which is less than 0.05. This means that an observed difference in these algorithms is significant. For the purpose of formally confirming which method is better, we conducted the Nemenyi test at the significance level 0.05 [31].

**Figure 6:**
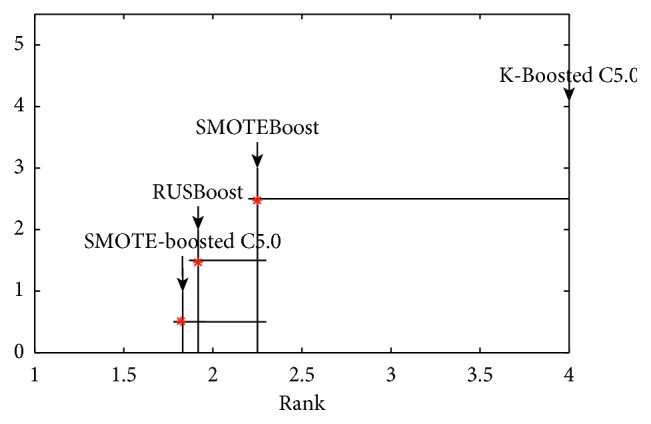
Results of the pairwise comparisons of methods using the Nemenyi post hoc test.

Figure 6 plots the methods according to their ranks. The “^*∗*^” denotes the respective average rank of each method, and the critical difference is represented by the line segment in its right. As it can be observed from the results of Figure 4 listed, the K-Boosted C5.0 method performs significantly better than other combinations for classification of imbalanced data sets.

## 4. Discussion

On the basis of our experimental analysis of the proposed method, following discussions are taken into consideration:

- MCC indicates that our proposed K-Boosted C5.0 approach is the best hybrid classifier for imbalanced datasets.
- In terms of accuracy, our proposed algorithm can maintain a good classification accuracy of overall class data except for the Redwine3 dataset. These improvements in classification accuracy are mainly due to the clustering-based undersampling method. For Redwine3, the best accuracy (89%) is obtained by the SMOTEBoost method which adds SMOTE into the boosting algorithms. In practice, SMOTE has good ability to balance dataset but how to choose sampling rate, which is crucial to its performance, and can be a time-consuming task. Thus, this fact restricts its use, and the performance is not stable. Experimental results show that the proposed K-Boosted C5.0 algorithm achieves relatively high, stable classification performance with less fixed parameters in most cases. So the proposed K-Boosted C5.0 is strongly desirable.
- In terms of *G*-mean, AUC, sensitivity, and specificity indicate that the proposed K-Boosted C5.0 exhibits unstable generalization. These are probably a consequence of different sensitivities of classifiers to various imbalance rates and other factors. Overall, we would indicate that our study considers all aspects of the imbalance problem, whereas the previous literature only focuses on accuracy.
- A comparison between using K-Boosted C5.0 and SMOTE-Boosted C5.0 over small-scale datasets shows that the proposed clustering-based undersampling method is better than SMOTE. Boosted C5.0 was validated by comparison with SVM and naive Bayes. This result can lead us to conclude that combining the clustering-based undersampling method with Boosted C5.0 provides the highest rate of classification MCC.
- To show the adaptation and generation capability of our proposed K-Boosted C5.0, we compare the results obtained by K-boosted C5.0 with the baseline approaches, RUSBoost and SMOTEBoost, over all the datasets. According to these results, the K-Boosted C5.0 delivers the optimal tested performance with the least amount of time, which was observed to be significantly different from the other (*p* < 0.05). It is interesting to note that the results of this paper are visible in medical field associate of ted with breast cancer disease and on large datasets. Note that according to the results of our experiment, the instance selection method which was proposed by Liu et al. [18], Chen et al. [17], and Lee et al. [8] in selecting the important instance can be resolved by applying cluster algorithms. In summary, the clustering-based undersampling is beneficial for instance selection. This finding provides more alternatives for selecting efficient instances classification models.
- Notably, Lin et al. [23] concluded that AdaBoost + C4.5 is the best ensemble classifier for breast cancer classification. Thus, in order to illustrate the good performance of the tree ensemble, we compare the results of K-Boosted C5.0, K-SVM, and K-NB. It is obvious from the results that the best classification MCC was obtained by the K-Boosted C5.0 method. This observation is consistent with the previous analyses. In addition, K-Boosted, K-SVM, K-NB, and RUSBoost are also compared over large-scale datasets, revealing the importance of the clustering-based undersampling method. Therefore, the above results demonstrate that the clustering-based undersampling method outperforms the other classical methods. This finding provides us another alternative as handling imbalanced classification problem.

From the experimental result on overall datasets, we found that, it is worth noting that K-Boosted C5.0 obtains the highest classification MCC but suffers from parameter setting, which is crucial to its classification performance. From a large body of the literature in breast cancer diagnosis, most methods are designed to maximize the overall classification accuracy only; not much work has been conducted for solving a breast cancer prediction task as a class imbalance problem. Actually, the accuracy, specificity, and sensitivity indices of the literature methods show controversial results on the breast cancer diagnosis. In addition, the accuracy of these classifiers is higher, yet lack specificity since the accuracy is overwhelmed by the instances in the majority class, by ignoring the instance in the minority class. Such imbalanced class distribution significantly hinders predictive performance and causes learning bias towards the majority class and leads toward poor generalization. Clustering technique groups the dataset into two clusters, and we select the informative majority and minority class instance from each cluster. With the help of clustering-based undersampling, the original data set is balanced. Our proposed K-Boosted C5.0 has shown its promising predictive performance in breast cancer diagnosis, balancing and remaining high MCC, and accuracy.

## 5. Conclusion and Future Work

In this paper, we propose a K-Boosted C5.0 algorithm based on undersampling to address the diagnosis of breast cancer and class imbalance problem. Our proposed method consists of two steps: firstly, K-means clustering is used to group the classes and find informative samples. We consider the instances which are close to the border of the cluster as the informative ones. We then set the distance parameter to make the majority and minority classes equal in number. Afterwards, Boosted C5.0 is performed for classification. Empirically, according to the experimental results, the K-Boosted C5.0 improves the performance significantly without increasing algorithm complexity. Furthermore, a clustering-based undersampling method actually provides a new way how to handle the class imbalance problem in an efficient manner.

A balanced, informative, and diverse training subset is obtained via k-means clustering in this work to encourage us to take this step further. In future, we would like to explore what are the effects of *η*, the parameter that can lead to improving the performance, in order to realize the importance of the number of instances in the training set that should be in consideration. We are also concerned with ensemble diversity that can enhance both overall and minority class performance.

## Figures and Tables

**Algorithm 1:**
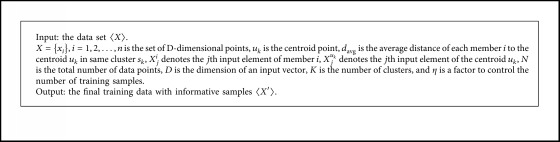
The clustering-based undersampling procedure.

**Table 1:** Experimental datasets.

Datasets	No. of data samples	No. of features	Imbalance ratio
Small-scale datasets			
(1) Abalone	731	8	16.4
(2) Bcwo	683	9	1.8577
(3) Pima	336	8	2.027
(4) Redwine1	837	11	3.21
(5) Redwine2	880	11	3.42
(6) Redwine3	734	11	12.85
(7) Redwine4	691	11	12.04
(8) Wbcd	569	30	1.8
(9) Whitewine	1043	11	5.4
(10) Yeast1	707	8	1.8975
(11) Yeast2	626	8	2.840
(12) Yeast3	892	8	1.08

Large-scale dataset			
(1) Breast cancer	102294	117	16319
(2) Protein homology prediction	145751	74	11146

**Table 2:** Confusion matrix.

	Predicted positive	Predicted negative
Actual positive	True positive (TP)	False negative (FN)
Actual negative	False positive (FP)	True negative (TN)

**Table 3:** Performance comparison based on Wbcd and Bcwo datasets.

Dataset	Method	Accuracy	Sensitivity	Specificity	*G*-mean	AUC	MCC
Wbcd	*K* + boosted C5.0	**0.982**	0.9375	**1**	**0.9682**	**0.969**	**0.956**
	SMOTEBoost	0.964	**0.946**	0.978	0.9619	0.963	0.924
	RUSBoost	0.944	0.93	0.954	0.942	0.942	0.886
	SMOTE-Boosted C5.O	0.925	0.939	0.911	0.9248	0.925	0.847

Bcwo	*K* + boosted C5.0	**0.975**	**0.991**	**0.969**	**0.9799**	**0.980**	**0.936**
	SMOTEBoost	0.92	0.98	0.89	0.934	0.933	0.839
	RUSBoost	0.936	0.926	0.944	0.9350	0.934	0.8539
	SMOTE-Boosted C5.0	0.937	0.934	0.941	0.9375	0.937	0.8756

**Table 4:** Performance comparison based on the Wbcd dataset.

ML method	Accuracy (%)	Sensitivity (%)	Specificity (%)	*G*-mean (%)	MCC
QKCLDA	97.26	—	—	—	
K-SVM	97.38	—	—	—	
PSO + Boosted c5.0	96.38	97.70	94.28	—	
Aisl	98.00	95.9	98.7	—	
PSO-KDE	98.45	100	97.99	—	
EC	96.5			—	
BBHA	97.38	95.79	98.57	—	
EM-PCA-CART-fuzzy					
Rule-based	93.2	—	—	—	
FSMLP	100	100	100	100	
**K-Boosted C5.0**	**98.2**	**93.75**	**100**	**96.82**	**95.6**

**Table 5:** Performance comparison based on the Bcwo dataset.

ML method	Accuracy (%)	Sensitivity (%)	Specificity (%)	*G*-mean (%)	MCC (%)
Aisl	98.3	94.3	99.6	96.91	
PSO-KDE	98.53	95.79	100	—	
**K-Boosted C5.0**	**97.48**	**1**	**96.17**	**98.07**	**93.6**

**Table 6:** Result comparison based on different datasets.

Dataset	Method	Accuracy	Sensitivity	Specificity	*G*-mean	AUC	MCC
Abalone	K-Boosted C5.0	0.960	0.2	0.992	0.445	0.596	**0.299**
	SMOTEBoost	0.822	0.628	0.834	0.724	0.730	0.264
	RUSBoost	0.592	0.802	0.58	0.682	0.69	0.15
	SMOTE-BoostedC5.0	0.618	0.635	0.601	0.618	0.624	0.232

Pima	K-Boosted C5.0	0.766	0.640	0.820	0.725	0.730	**0.454**
	SMOTEBoost	0.75	0.646	0.8	0.719	0.723	0.432
	RUSBoost	0.714	0.792	0.676	0.732	0.733	0.446
	SMOTE-BoostedC5.0	0.713	0.742	0.684	0.712	0.713	0.425

Redwine1	K-Boosted C5.0	0.823	0.517	0.905	0.684	0.823	**0.446**
	SMOTEBoost	0.784	0.544	0.858	0.683	0.701	0.397
	RUSBoost	0.702	0.656	0.718	0.686	0.69	0.34
	SMOTE-BoostedC5.0	0.688	0.715	0.661	0.687	0.688	0.377

Redwine2	K-Boosted C5.0	0.902	0.681	0.969	0.812	0.825	**0.712**
	SMOTEBoost	0.826	0.85	0.82	0.835	0.835	0.59
	RUSBoost	0.82	0.852	0.812	0.832	0.832	0.577
	SMOTE-BoostedC5.0	0.844	0.865	0.822	0.8432	0.843	0.691

Redwine3	K-Boosted C5.0	0.834	0.410	0.866	0.596	0.637	**0.194**
	SMOTEBoost	0.89	0.12	0.948	0.337	0.545	0.054
	RUSBoost	0.764	0.46	0.788	0.602	0.634	0.171
	SMOTE-BoostedC5.0	0.567	0.509	0.625	0.564	0.573	0.137

Redwine4	K-Boosted C5.0	0.940	0.263	0.989	0.510	0.626	**0.38**
	SMOTEBoost	0.916	0.28	0.964	0.520	0.623	0.317
	RUSBoost	0.678	0.64	0.682	0.661	0.660	0.152
	SMOTE-BoostedC5.0	0.618	0.635	0.601	0.618	0.624	0.232

Whitewine	K-Boosted C5.0	0.925	0.650	0.961	0.79	0.805	**0.625**
	SMOTEBoost	0.804	0.838	0.798	0.818	0.818	0.502
	RUSBoost	0.794	0.85	0.784	0.816	0.817	0.49
	SMOTE-BoostedC5.0	0.796	0.801	0.792	0.796	0.797	0.593

Yeast1	K-Boosted C5.0	0.952	0.957	0.949	0.953	0.957	**0.898**
	SMOTEBoost	0.762	0.722	0.788	0.754	0.754	0.497
	RUSBoost	0.798	0.694	0.852	0.769	0.773	0.552
	SMOTE-BoostedC5.0	0.723	0.734	0.712	0.723	0.723	0.45

Yeast2	K-Boosted C5.0	0.951	0.924	0.958	0.941	0.941	**0.855**
	SMOTEBoost	0.932	0.904	0.94	0.922	0.921	0.836
	RUSBoost	0.93	0.938	0.926	0.928	0.931	0.824
	SMOTE-BoostedC5.0	0.9116	0.8934	0.9388	0.9158	0.916	0.821

Yeast3	K-Boosted C5.0	0.646	0.575	0.706	0.637	0.641	**0.28**
	SMOTEBoost	0.618	0.618	0.612	0.615	0.616	0.232
	RUSBoost	0.64	0.544	0.728	0.629	0.636	0.27
	SMOTE-BoostedC5.0	0.598	0.450	0.735	0.575	0.593	0.195

**Table 7:** Mean rank of the Friedman test over the four classification algorithms.

*p*-value	K-Boosted C5.0	SMOTEBoost	RUSBoost	SMOTE-BoostedC5.0
7.488*e* − 08	4	2.25	1.917	1.83

## Data Availability

The datasets in these experiments are taken from the public UCI machine learning repository.
